# A Drab, Inexpressibly Dreary Neighbourhood

**Published:** 1917-10-27

**Authors:** 


### "A Drab, Inexpressibly Dreary Neighbourhood."

The appeal of the Rev. H. W. G. Kenrick, M.A., Vicar of Holy Trinity, Shepherdess Walk, Hoxton, is one to win sympathy and support. Here it is:

Among the many miseries which the war entails is the plight of an East End parish, where the ordinary flow of money and gifts has almost, if not entirely, ceased. The position of Holy Trinity, Hoxton, situated in a drab, inexpressibly dreary neighbourhood, is one of the deepest anxiety. The war has greatly affected the financial position of many who have hitherto helped us, and upon whom we rely for the means to carry on the work among 7,000 people. There is no local help, and the need for the church is great. We have a Relief Committee, Lads' Brigade, Sunday Schools, Mothers' Meetings, Guilds, Clubs, and many services, but the whole burden of providing the funds falls upon the Vicar, and only those who live in Hoxton can realise what that means. There are calls which naturally appeal strongly to people just now, but I plead for the aged, the sick, and the children, that they may not be forgotten, and I ask that even in these times of stress and unprecedented demands upon your purse and sympathy that you will send me a donation, however small, for the love of God and for the sake of those so much less fortunate than yourselves.

There are, no doubt, among the readers of The Hospital several at least who could send a contribution to Mr. Kenrick, and we hope that every one of them will do so quickly.

